# C-Reactive Protein and Neurological Autoimmune Diseases: Bridging the Diagnostic and Pathogenic Gap

**DOI:** 10.3390/ijms27031322

**Published:** 2026-01-28

**Authors:** Patrik Buzgau, Mark Slevin, Ioana Theodora Barna, Lóránd Dénes, Amelia Tero-Vescan, Aurelio Pio Russo, Ylenia Pastorello

**Affiliations:** 1 Faculty of Medicine, George Emil Palade University of Medicine, Pharmacy, Science and Technology of Târgu Mureș, 540139 Târgu Mureş, Romania; patrik.buzgau@umfst.ro; 2 Centre for Advanced Medical and Pharmaceutical Research, George Emil Palade University of Medicine, Pharmacy, Science and Technology of Târgu Mureș, 540139 Târgu Mureş, Romania; 3 Faculty of Dentistry, George Emil Palade University of Medicine, Pharmacy, Science and Technology of Târgu Mureș, 540139 Târgu Mureş, Romania; barna.ioana-theodora.21@stud.umfst.ro; 4 Department of Anatomy and Embryology, George Emil Palade University of Medicine, Pharmacy, Science and Technology of Târgu Mureș, 540139 Târgu Mureş, Romania; lorand.denes@umfst.ro (L.D.); ylenia.pastorello@gmail.com (Y.P.); 5 Department of Medical Chemistry and Biochemistry, George Emil Palade University of Medicine, Pharmacy, Science and Technology of Târgu Mureș, 540139 Târgu Mureş, Romania; amelia.tero-vescan@umfst.ro; 6 Doctoral School of Medicine and Pharmacy, George Emil Palade University of Medicine, Pharmacy, Science and Technology of Târgu Mureş, 540139 Târgu Mureş, Romania; aurelio.russo@umfst.ro

**Keywords:** C-reactive protein, inflammation, autoimmune diseases, multiple sclerosis, neuromyelitis optica spectrum disorder, myasthenia gravis, Guillan–Barré

## Abstract

C-reactive protein (CRP) has emerged as a crucial link between systemic and neuroinflammatory processes, though its role across neurological autoimmune disorders remains incompletely understood. Pathologies such as multiple sclerosis (MS), neuromyelitis optica spectrum disorder (NMOSD), Guillain–Barré syndrome (GBS), and myasthenia gravis (MG) share chronic, dysregulated inflammation resulting from loss of immune tolerance. Their pathogenesis arises from interactions among genetic susceptibility, environmental factors, and gut microbiota alterations that trigger autoreactive immune cascades through molecular mimicry, ectopic antigen expression, or paraneoplastic cross-reactivity. These immune pathways sustain inflammation and promote neuroaxonal injury. CRP, synthesized mainly by hepatocytes in response to interleukin-6 (IL-6), functions as both an effector and reporter of inflammation, linking systemic immune activation to neuroinflammatory damage. Elevated CRP levels correlate with unfavorable outcomes, including accelerated disability in MS, IL-6-mediated astrocyte injury in NMOSD, respiratory failure in GBS, and crisis susceptibility in MG. Composite indices such as the CRP-to-albumin ratio are emerging as refined prognostic markers, though interpretation is limited by non-specificity and biological variability. This review integrates current evidence on CRP’s mechanistic roles, clinical associations, and translational potential in neuroinflammatory disorders, combining molecular, clinical, and imaging perspectives to refine its role within inflammation-driven neurodegeneration.

## 1. Introduction

While there is currently no clear consensus on the specific pathologies that define autoimmune diseases or on their classification [1], the fundamental understanding of their pathogenesis centers on dysfunctional mechanisms governing the elimination or regulation of self-reactive lymphocytes [2]. Although only a small fraction of immune responses are directed against autologous tissues, autoimmunity now encompasses over 80 to 100 distinct diseases [3], and is estimated to affect approximately 6–8% of the global population. The initiation of autoimmunity is attributed to a combination of genetic susceptibility through polymorphism, environmental triggers, and impaired immunoregulatory mechanisms, acting in sequential mechanisms ending either in resolution or recrudescence [1,4,5]. Inflammation serves as both a mediator and a consequence of this dysregulation. Under physiological conditions, it is a controlled biological response aimed at injury containment and tissue repair. In autoimmune diseases, however, prolonged immune activation transforms this protective process into a self-perpetuating pathological cycle. Persistent stimulation of innate immunity, through cytokines such as interleukin-1β (IL-1β), interleukin-17 (IL-17), tumor necrosis factor-α (TNF-α), and interferons, disrupts tolerance by continuously activating adaptive immune cells [6]. The resulting interplay between innate and adaptive pathways, amplified by mediators like C-reactive protein (CRP), propagates tissue injury and sustains disease chronicity. Thus, inflammation not only reflects the effector phase of autoimmunity but also constitutes its driving force, unifying immune dysregulation and pathological progression within a single continuum [7]. While existing treatment modalities frequently emphasize attenuation of inflammatory processes, such approaches may not be universally effective across patient populations [6].

The objective of this review is to provide a state-of-the-art understanding of the pathophysiological mechanisms interlinking inflammation and autoimmunity, as well as their diagnostic and prognostic relevance, given their fundamental importance in the development of future targeted therapeutics.

## 2. Inflammatory Pathways in Neurological Autoimmune Disorders

### 2.1. Autoimmune Disorders of the Nervous System

Autoimmune disorders of the nervous system comprise a clinically and pathologically diverse group of diseases that can affect both central and peripheral structures, exhibiting distinct yet interrelated immunopathogenic mechanisms and varied clinical presentations. These conditions include multiple sclerosis (MS), neuromyelitis optica spectrum disorder (NMOSD), and autoimmune encephalitis within the central nervous system (CNS), and disorders such as Guillain–Barré syndrome (GBS), chronic inflammatory demyelinating polyneuropathy, and myasthenia gravis (MG) in the peripheral nervous system (PNS). Autoimmune diseases may target specific neural antigens, as illustrated by the aquaporin-4 IgG autoantibodies in NMOSD or acetylcholine receptor autoantibodies in MG, resulting in a spectrum of immune-mediated tissue injury ranging from demyelination and axonal degeneration to synaptic dysfunction [8,9].

In MS, immune activation, marked by perivascular lymphocyte infiltration and macrophage-driven myelin destruction, leads to neurological impairment that frequently follows a relapsing-remitting or progressive course. Epidemiologically, MS exhibits a marked female-to-male predominance and demonstrates significant geographical and genetic determinants modulating disease risk and expression [10,11,12]. NMOSD was only recently distinguished from MS, largely based on specific autoantibody profiles and clinical features, with astrocytopathy and demyelination causing recurrent optic neuritis and longitudinally extensive transverse myelitis. Targeted immunotherapies have significantly improved the management of both acute relapses and chronic disability [13,14,15].

Peripheral nervous system involvement is exemplified by GBS, an acute, frequently post-infectious polyradiculoneuropathy characterized by rapid, symmetrical muscle weakness and areflexia. Its pathogenesis is uniquely associated with antibody-mediated injury to myelin or axonal components, precipitated by molecular mimicry. GBS shows age- and sex-dependent epidemiological trends, being predominant in young women and more common in older men [16,17,18]. MG, the most common autoimmune disorder of the neuromuscular junction, presents with episodic muscle weakness linked to autoantibody-driven loss of acetylcholine receptors and complement-mediated disruption of postsynaptic membrane integrity. The spectrum extends from mild ocular symptoms to life-threatening respiratory failure, and electrophysiological abnormalities provide objective confirmation of neuromuscular transmission deficits [8,19].

Despite the diversity in targeted antigens, affected anatomical regions, and clinical manifestations, all these diseases share a unifying pathogenic theme: the breakdown of immune tolerance and subsequent chronic or relapsing inflammation, sustained by dysregulated innate and adaptive immune responses. Advances in immunological techniques have enabled earlier diagnosis and more tailored therapeutic approaches, aiming to suppress aberrant immune activation, limit irreversible neurological damage, and improve long-term outcomes [8,16,17].

### 2.2. Role of Inflammation in Neurological Autoimmune Diseases

Inflammatory mechanisms in autoimmunity are primarily centered around the breakdown of immunological tolerance, the ectopic expression of self or onconeural antigens, and external environmental factors such as infections and alterations in the gut microbiota. These elements contribute to the activation of autoreactive lymphocytes and the perpetuation of chronic inflammation within target tissues [20]. Numerous infections have been implicated in the subsequent development of MS in animal models, suggesting a potential role for infectious agents as environmental triggers in the initiation or exacerbation of autoimmune demyelination, namely human herpesvirus-6 (HHV-6) and Epstein–Barr virus (EBV) [21]. A prior diagnosis of infectious mononucleosis, typically caused by EBV, has been consistently associated with an increased risk of developing MS. Conversely, EBV seronegativity is strongly correlated with a markedly reduced likelihood of MS, suggesting a critical role for EBV in disease pathogenesis [22].

Molecular mimicry is considered one of the principal mechanisms through which infectious agents or chemical exposures may trigger autoimmunity. This process occurs when structural similarities between foreign and self-peptides lead to the activation of autoreactive T or B lymphocytes by a foreign-derived antigen in genetically susceptible individuals [23]. Molecular mimicry is a well-established pathogenic mechanism in GBS, particularly in cases triggered by prior infections. GBS is often preceded by bacterial or viral infections, most notably *Campylobacter jejuni*, which is strongly associated with the acute motor axonal neuropathy (AMAN) variant where between bacterial lipooligosaccharides showcase similarities to gangliosides expressed on peripheral nerve axons and Schwann cells resulting in cross reactive antibodies. These antibodies, such as anti-GM1, anti-GD1a, or anti-GQ1b, bind to neuronal gangliosides, activating complement and leading to demyelination or axonal degeneration. The resulting immune-mediated damage manifests clinically as rapidly progressive weakness and areflexia [24].

Paraneoplastic cerebellar degeneration (PCD) is a rare autoimmune neurological disorder characterized by progressive cerebellar dysfunction, including ataxia, dysarthria, and nystagmus. It is most commonly associated with underlying malignancies such as breast, ovarian, and small cell lung carcinomas. The condition is frequently linked to the presence of anti-Yo, anti-Hu, or anti-Tr antibodies, which target cerebellar Purkinje cells. Similarly, limbic encephalitis, another paraneoplastic syndrome, is often associated with small cell lung carcinoma (SCLC) and is mediated by antibodies such as anti-Hu or anti-Ma2. This disorder affects the medial temporal lobes and presents with symptoms such as memory loss, seizures, and psychiatric disturbances. In both conditions, immune responses directed against tumor-expressed neuronal antigens result in cross-reactive inflammation and neuronal damage within the CNS [25].

Emerging evidence increasingly implicates the gut–brain axis in MS pathogenesis. In a study of 34 identical twin pairs where one twin had MS and the other did not, researchers found no major overall differences in gut microbes. However, certain bacteria like Akkermansia were more abundant in untreated MS-affected twins. When gut microbiota from these MS twins were transplanted into genetically predisposed mice, the mice developed autoimmune brain inflammation more frequently than those receiving microbiota from healthy twins [26].

### 2.3. C-Reactive Protein and Its Activity in the Inflammatory Response

Representing one of only two existing short pentraxins in the human body, CRP is a pentameric acute-phase protein with foundational implications in innate immunity. It functions in a dual, often context-dependent role: accelerating the clearance of pathogens and cellular debris while simultaneously promoting immunological self-limitation. In the presence of calcium, CRP undergoes a conformational change that allows it to bind with high affinity to phosphocholine (PC) moieties. While these moieties are hallmarks of bacterial polysaccharides, they are also prominently exposed on the damaged membranes of neurons and myelin sheaths during neuroinflammatory insults. Once anchored to these damaged surfaces, CRP acts as a molecular bridge, binding to Fcγ cell surface receptors on microglia and infiltrating macrophages, significantly enhancing the kinetics of phagocytosis. More importantly, once CRP is bound to its ligand, it provides a high-affinity docking site for the complement component 1q (C1q), the initiating protein of the classical complement pathway. In the context of the CNS, this interaction is represented by a double act. The recruitment of C1q leads to the formation of the membrane attack complex (MAC), which may exacerbate myelin breakdown and axonal instability in diseases like MS [27,28,29].

Protective opsonization facilitates the rapid clearance of myelin debris, a prerequisite for eventual remyelination. To prevent runaway inflammation, CRP-mediated activation is coupled with the recruitment of Factor H, a key regulator that inhibits the alternative complement pathway. This balanced recruitment ensures that while damaged structures are targeted for removal, “bystander” damage to healthy, intact neural tissue is carefully regulated [30,31].

CRP exists in the body in two main forms, pentameric CRP (pCRP) which is the native circulating form primarily synthesized in the liver, while monomeric CRP (mCRP) disassociated from it in inflammatory scenarios. mCRP functions by increasing neutrophil adhesion to endothelial cells (ECs), via IL-8, intercellular adhesion molecule 1 (ICAM-1) and vascular cell adhesion molecule (VCAM) [32,33]. In neurodegenerative pathology, mCRP is known to promote cerebrovascular inflammation and vascular proliferation, activating microglia, macrophages and neurons via inducible nitric oxide synthase (iNOS) and cyclooxygenase-2 (COX-2) pathways [34]. Its role as an inflammatory trigger can be traced to patients suffering from cerebral ischemia as well as coexisting with B amyloid or p-Tau neurons exacerbating existing inflammation and neurodegenerative processes [34,35]. Direct quantification of mCRP in cerebrospinal fluid (CSF) remains technically and clinically underdeveloped. To date, there are no validated clinical assays or published feasibility studies demonstrating reliable mCRP detection in CSF from patients with neuroinflammatory diseases such as MS or GBS. Although mCRP has been localized to neuroinflammatory regions of the brain, particularly following ischemic stroke and in Alzheimer’s disease (AD), its detection has relied primarily on immunohistochemical approaches rather than fluid-based assays. These studies show mCRP association with activated glial cells, infiltrating macrophages, and areas of blood–brain barrier (BBB) disruption.

Both serum and CSF mCRP measurement are technically possible in research settings, but both matrices have important limitations that currently prevent routine clinical deployment in neuroinflammatory disease. Serum and plasma assays are the closest to clinical feasibility because conformation-specific enzyme-linked immunosorbent assays (ELISA) exist, yet pre-analytical and analytical issues still complicate interpretation and inter-lab reproducibility [36]. The distinct biochemical profile of CSF specifically its low total protein concentration and unique lipid composition presents significant analytical hurdles, including potential matrix interference and analyte loss due to surface adsorption. Consequently, standard assays may require substantial optimization to ensure sensitivity and accuracy in this medium [36,37]. Furthermore, interpreting CSF mCRP levels is complicated by the potential for passive diffusion from systemic circulation; an elevated signal may reflect increased BBB permeability rather than primary neuroinflammation or intrathecal synthesis. Importantly, the transition of pCRP to its monomeric isoform is often triggered by acidic microenvironments or contact with activated membranes. This suggests that the exogenous conditions of CSF collection and processing such as surface contact or altered ionic strength could theoretically induce pCRP dissociation, leading to the measurement of artifactual mCRP that does not reflect the in vivo state [38,39].

Serum mCRP measurement is more clinically feasible than CSF due to established conformation-specific sandwich ELISAs yet faces similar limitations including incomplete standardization across labs, risk of pCRP cross-reactivity masking low-level signals, variability from microparticle/vesicle binding during handling, poor correlation with total pCRP levels, and substantial protein losses in purification steps that undermine reproducibility [40].

### 2.4. C-Reactive Protein’s Relevancy and Clinical Role

Historically, CRP has not always been the most prominent biomarker for reflecting inflammation. Other well researched acute-phase proteins also exhibit distinct changes in plasma concentration during the acute-phase response such as serum amyloid A (SAA) or albumin [41].

Elevated CRP has been associated with increased risk of death from cardiovascular disease and stroke, the development of AD and dementia, and rheumatologic diseases such as systemic lupus erythematosus (SLE) [42,43].

There is evidence of mice receiving temporary protection from SLE after injection with human CRP, the temporary effect being attributed to the development of an immune response against the externally injected agent [28].To circumvent that, transgenic mice (CRPtg) that express human CRP as an acute-phase protein were developed using a human transgene, reaching blood concentrations comparable to those observed in humans [44]. Research conducted by Szalai [27], used this premise to investigate if human CRP provides systemic protection against SLE and MS. In mouse models, CRP expression delayed disease progression, reduced proteinuria and renal deposits, and suppressed pro-inflammatory cytokine production via IL-10. These protective effects are attributed to CRP’s role in modulating complement and Fcγ receptors (FcγRs) and its ability to bind to apoptotic cells and autoantigen. Collectively, these transgenic and passive-transfer studies suggest that CRP could go from a simple bystander to an active regulator of immune tolerance, increasing potential therapeutic opportunities for modulating CRP pathways in autoimmune conditions such as SLE and MS.

While CRP is a recognized marker of systemic inflammation, its utility as a specific biomarker for MS, NMOSD, or CNS lupus remains limited. Standard assays lack the specificity to differentiate these conditions, and a scarcity of longitudinal data obscures the relationship between CRP fluctuations and ‘silent’ disease progression. Furthermore, the reliance on total serum CRP often overlooks the potential of high-sensitivity CRP (hs-CRP) to detect subclinical inflammation or the diagnostic precision offered by localized CSF-CRP levels [38,45,46]. Thus, in diseases like MS, NMOSD, and MG, mCRP holds research promise for reflecting localized neuroinflammation, however with important caveats in clinical adoption without validated protocols in distinguishing peripheral leakage [9,37,47,48].

### 2.5. CRP, the Blood–Brain Barrier, and the Limits of Systemic Inflammatory Biomarkers in Neuroinflammatory Disease

CRP does not freely traverse the BBB and lacks a known saturable transport mechanism with detectable central levels being typically associated with BBB disruption permitting passive leakage [49]. However, in early neurodegeneration or in CNS inflammatory diseases such as MS with relatively limited inflammatory activity, BBB alterations are often subtle and may restrict permeability to the extent that only minimal, clinically insignificant passage of large plasma proteins occurs [50]. Nevertheless, peripheral immune signals can modulate CNS function through several convergent pathways. Circulating cytokines triggered by systemic inflammation may access the brain at regions with a permeable BBB, such as the circumventricular organs [46], or undergo active, saturable transport across the BBB [47]. In addition, cytokines can act at the neurovascular unit, stimulating cerebral ECs and perivascular macrophages to generate local inflammatory mediators, including prostaglandins, nitric oxide (NO), and secondary cytokines, thereby amplifying CNS immune signaling without direct barrier penetration [37]. It is important to note that local generation of mCRP can occur in the CNS as a consequence of vascular-mediated inflammation, with astrocytes, microglia, and neurons responding to classical acute-phase signaling pathways [51,52]. Local mCRP deposits observed in neurodegenerative disorders and following vascular infarction have traditionally been interpreted as pathogenic; however, they may also be viewed as secondary contributors that amplify subsequent inflammatory cascades and promote progressive neurodegeneration [36]. The pathogenic gap appears as elevated CRP levels are not reliably associated with intrathecal inflammation in neuroinflammatory diseases. In MS, intrathecal immune activity is more accurately characterized by CSF-specific markers, including oligoclonal bands, IgG index, and CSF cytokine profiles [47]. In GBS, although CRP levels may correlate with clinical severity and overall systemic inflammatory burden, CNS involvement is better reflected by alternative biomarkers rather than CRP [53]. Similarly, in NMSODs, despite the demonstrated clinical relevance of specific cytokines, CRP remains an unreliable indicator of intrathecal inflammation [54].

### 2.6. Metabolic Syndrome, and Other Cofounding Variables in Neuroinflammatory Disease

The relationship between obesity and CRP in neuroimmunological disorders may extend beyond simple confounding. Obesity and metabolic syndrome are characterized by chronic low-grade systemic inflammation and increases in leptin values with structural similarity with Th17 cells, central to NMOSD and MS pathogenesis [55,56,57]. Elevated CRP in such conditions reflects inflammation from adipose-derived cytokine activity as well as aquaporin-4 (AQP4)-immunoglobulin G (IgG) activity in NMOSD for example. Several immunosuppressive therapies used for neuroinflammatory autoimmune disease can also induce iatrogenic weight gain and insulin resistance, contributing to the list of confounding variables in relation to CRP activity [58]. Individuals with metabolic syndrome consistently demonstrate higher CRP levels compared with metabolically healthy controls, and central obesity and BMI are major determinants of this elevation [59,60]. Adjustment for adiposity often diminishes the association between metabolic syndrome and CRP, with both factors possibly even contributing to disease progression [61]. Cytokine activity in patients with increased adiposity has shown to modify BBB permeability by reducing the production of junction proteins and promoting endothelial dysfunction by modulating NO production [58]. Similar considerations apply to other antibody-mediated neuroimmunological disorders, including NMOSD and MG, where CRP is not a disease-specific biomarker and may be influenced by metabolic dysregulation or treatment-related factors such as long-term corticosteroid exposure [37,48,62].

## 3. CRP Levels in Neurological Autoimmune Diseases

### 3.1. CRP in Multiple Sclerosis

Over 150 years ago, Charcot’s seminal lectures first described MS as a distinct condition of the CNS, in association with neuropsychiatric symptoms such as euphoria, anxiety and major depression as foundational characteristics [63]. More recent epidemiological data confirms a significantly higher rate of experiencing major depression in adults with MS rather than the general population, approaching 30–50% in studied cohorts. With both anxiety and depression often co-occurring as internalizing pathologies in MS, their combined presence has become a predictor of cognitive impairment for such patients [58,64].

Evidence suggests that systemic inflammation is an important driver in the pathophysiology of depression. Cardinal symptoms such as cognitive dysfunction mentioned earlier were associated with elevated cytokine and acute phase protein activity. These cytokines act as a bridge between the immune system and the central nervous system, disrupting the hypothalamic–pituitary–adrenal (HPA) axis by influencing corticotropin-releasing hormone (CRH) and cortisol secretion [55]. Furthermore, chronic cytokine exposure impairs synaptic plasticity by reducing brain-derived neurotrophic factor (BDNF) and alters serotonin metabolism, creating a biochemical environment conducive to depression [54,56,57]. In the young population, elevated CRP levels are also predictive of later depression severity, and antidepressants have been shown to interact with these values [59,60,61,62]. Similarly, anxiety correlates with increased CRP concentrations [65]. In the context of MS, this inflammatory environment coincides with specific structural damage. Lesion burdens within fronto-limbic tracts and “depression networks” correlate directly with symptom severity, suggesting a specific neuroanatomical link between MS-related white matter damage and emotional dysregulation [66,67,68].

### 3.2. CRP in Neuromyelitis Optica Spectrum Disorder

NMOSD, initially termed Devic’s disease or neuromyelitis optica (NMO), was first described in the 19th century in patients presenting with concurrent acute myelitis and optic neuritis [69]. While Devic and Gault believed that there was enough reasoning to separate MS and NMO, other authors disagreed with no clear consensus until the 2004 discovery of pathogenic antibodies specific to AQP4 [69]. Both exhibit overlapping clinical presentations, including optic neuritis and transverse myelitis, as well as mechanisms of demyelination, neuroaxonal injury, and BBB disruption. The concept of spectrum as opposed to a singular disease has been reinforced by evidence showing that some patients with clinical features of NMOSD such as myelitis and optic neuritis, may instead have these symptoms as manifestations of other underlying conditions, including connective tissue diseases, paraneoplastic syndromes, or infections [9].

In NMOSD, IL-6 plays a central pathogenic role by promoting the differentiation of pro-inflammatory T-helper 17 (Th17) cells and enhancing the secretion of pathogenic AQP4-IgG. IL-6 also disrupts the BBB, facilitating the entry of autoantibodies and immune cells into the CNS. This cascade contributes indirectly to astrocyte injury, followed by secondary demyelination and neuronal damage [13]. Thus, the hypothesis of CRP maintaining a similar clinical utility in NMOSD warrants further exploration, given the connection between IL-6 and CRP.

As an important regulator in glutathione metabolism and abundant resource on surface cerebral capillaries, gamma-glutamyl transferase (GGT) is an enzyme in maintaining BBB integrity by managing oxidative stress [70,71]. As such, a permeable or dysfunctional BBB will permit indirect CRP passage into the CNS, with GGT representing a useful indicator in monitoring BBB integrity. The work of Shu et al. confirmed this hypothesis, with NMOSD patients exhibiting increased CRP and GGT levels, further expanding the role of these substances as biomarkers in neuroinflammation [72].

**Figure 1:**
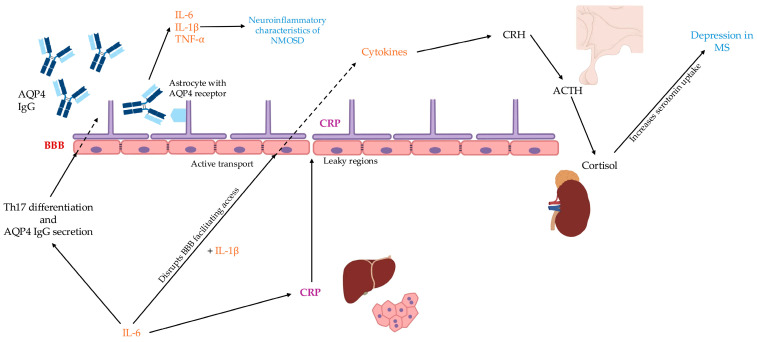
In this model, IL-6 drives hepatic CRP production and stimulates the release of IL-1β which is known to compromise BBB integrity. It is suggested that cytokines may then enter the CNS via active transport or through regions of increased permeability (dashed arrow). Once in the CNS, these cytokines are hypothesized to upregulate CRH production, triggering a cascade of increased ACTH and subsequently elevated cortisol. While the link between cortisol and mood is complex, it is argued that elevated cortisol may enhance serotonin uptake, potentially contributing to the depressive symptoms observed in MS. In the specific context of NMOSD, IL-6 further drives Th17 cell differentiation and enhances the secretion of pathogenic AQP4-IgG. While IL-6 is an established disruptor of the BBB, it is proposed that CRP may collectively contribute to this permeability, enabling the infiltration of autoantibodies and immune cells (dashed arrow). This cascade culminates in astrocyte injury, with secondary demyelination and neuronal loss following as downstream consequences.

The pathophysiological mechanisms involved in MS and NMOSD are presented in Figure 1.

### 3.3. CRP in Guillan–Barré Syndrome

Autoantibodies in Guillain–Barré Syndrome bind to peripheral nerve components, initiating the classical complement cascade on the nerve surface. This activation leads to the deposition of complement products that directly damage nerve membranes and attract macrophages, amplifying inflammation and tissue destruction. The formation of the membrane attack complex causes cell lysis and nerve fiber injury, while the release of anaphylatoxins further promotes neuroinflammation by activating macrophages and other immune cells within the nerve, perpetuating ongoing damage [17]. Disruption of the blood-nerve barrier increases its permeability, permitting circulating antibodies and immune cells to infiltrate the nerve tissue, which amplifies inflammation and contributes to nerve damage [16]. In GBS, elevated CRP levels are frequently detected, reflecting the heightened systemic inflammation characteristic of the disease. Higher CRP concentrations have been associated with greater disease severity, increased neurological deficits, and adverse outcomes, including the need for mechanical ventilation and a poorer overall prognosis [16].

Approximately 80% of patients with GBS reach the clinical nadir within two weeks of symptom onset, and nearly all patients do so within four weeks [18].

In the study conducted by Vaishnavi et al. [73], CRP levels were measured using both a semi-quantitative latex agglutination test (LAT) and ELISA. CRP levels reached up to 19 mg/dL when measured by both LAT and ELISA methods. Additionally, the rate of CRP positivity was higher with ELISA compared to LAT. CRP measured by LAT was positive in 24.4% of the GBS group, 34% of the non-paralytic neurological symptoms/disorder group, and 44% of the non-neurological symptoms) group. Among CRP-positive samples, titers ranged from 0.6 mg/dL to 19.2 mg/dL across all three patient groups. Comparable results were obtained using ELISA. In contrast, none of the healthy control subjects showed detectable CRP levels. Notably, elevated basal CRP levels were observed in patients with GBS. With the link between GBS and elevated CRP established, the question remains about possible correlations between disease progression, clinical manifestations and CRP levels.

### 3.4. CRP in Myastenia Gravis

Significant progress in the understanding of MG occurred in the 20th century, notably in the 1930s, when Dr. Mary Walker demonstrated that muscle weakness in MG patients could be temporarily alleviated by physostigmine, a cholinesterase inhibitor, thereby implicating the neuromuscular junction in the disease process. The autoimmune etiology of MG was later confirmed in the 1970s with the identification of autoantibodies directed against acetylcholine receptors (AChRs) on the postsynaptic membrane, elucidating a key pathogenic mechanism [19]. Indeed, autoantibodies directed against nicotinic AChRs at the neuromuscular junction inhibit receptor function, facilitate receptor internalization and degradation, and trigger complement-mediated damage. This loss of functional AChRs disrupts postsynaptic depolarization and sodium-dependent muscle contraction, resulting in muscle weakness [8].

Recent research has associated MG with elevated levels of inflammatory proteins, notably matrix metalloproteinase-10 (MMP-10), suggesting a potential role in disease pathogenesis [74]. Metalloproteinases are zinc-dependent enzymes that degrade extracellular matrix components and are involved in physiological processes such as tissue remodeling and inflammation. Dysregulation of these enzymes, particularly matrix metalloproteinases (MMPs), contributes to pathological conditions including neuroinflammation and BBB disruption [75]. The pro-inflammatory milieu evidenced in MG is the key precursor of augmented levels of CRP encountered in MG patients [48]. In this context, Cimmino et al. tested the link between MMPs, specifically matrix metalloproteinase-9 (MMP-9), and CRP both in vitro and in vivo. Respectively, CRP dose-dependently induced MMP-9 expression in smooth muscle cells (SMCs) by promoting MMP-mRNA transcription, as well as MMP-9 secretion, and a significant correlation between MMP-9 and CRP plasma levels was found in patients with acute coronary syndromes [76]. Therefore, further stimulation of MMP activities by CRP may create a plausible reinforcing loop which exacerbate tissue injury, disease progression and clinical decline.

Using whole-transcriptome sequencing of blood cells from AChR antibody-positive early-onset MG patients, researchers identified numerous dysregulated coding genes and microRNAs (miRNAs) associated with inflammatory and infectious pathways. Notably, key genes like interleukin-4 (IL-4), protein phosphatase 1 regulatory subunit 15A (PPP1R15A), and others were altered, with IL-4 downregulation reinforcing its suspected protective role in MG [77].

MG is frequently associated with abnormalities of the thymus, most commonly thymic hyperplasia and, in some cases, thymoma [77]. Thymic hyperplasia, characterized by the presence of germinal centers, is observed in approximately 65% of MG patients, particularly in younger individuals, and reflects an active autoimmune response within the thymus. Thymomas, which are tumors of the thymic epithelial cells, occur in about 10–15% of MG cases, typically in older adults. These tumors may be benign or malignant and are strongly linked to autoimmune diseases, including MG. Due to this strong association, imaging studies of the anterior mediastinum, such as computed tomography (CT) or, magnetic resonance imaging (MRI) are routinely performed in patients diagnosed with MG to assess for thymic abnormalities [78]. These immune-related changes persisted despite thymectomy or immunosuppressive treatment, indicating ongoing disease activity. Additionally, novel miRNAs such as miR-612 and their target genes (e.g., histamine H4 receptor (HRH4) and A-kinase anchoring protein 12 (AKAp12)) may play regulatory roles in the autoimmune process [79].

Anti-CD20 and IL-6 receptor-targeted therapies profoundly reduce baseline CRP by depleting antibody-producing cells and suppressing cytokine signaling, which can mask CRP elevations associated with myasthenic crises or relapses. Similarly, corticosteroids and rituximab can normalize CRP levels even in the presence of active autoimmunity [80]. Therefore, disease activity and relapse in MG must be monitored using clinical scales such as MG activities of daily living (MG-ADL) or quantitative MG (QMG), AChR antibody titers, or electrophysiological studies such as single-fiber EMG, rather than relying on CRP trends [81].

**Figure 2:**
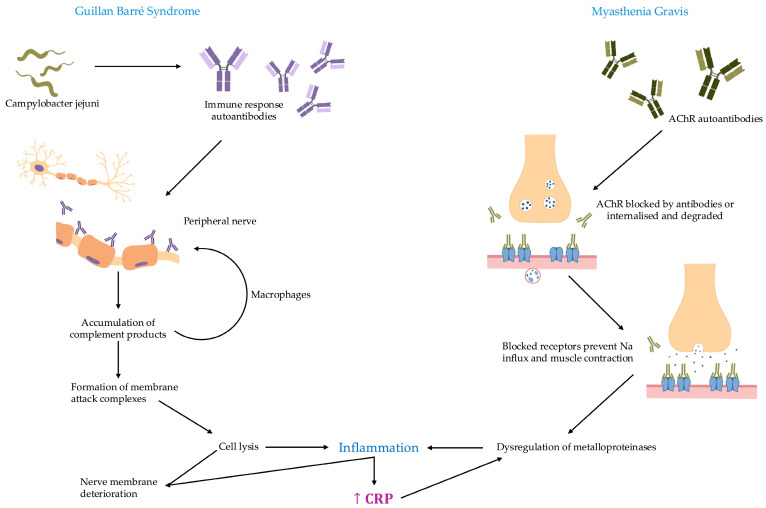
In GBS, antibodies induced by *Campylobacter jejuni* bind to peripheral nerves, triggering the classical complement cascade. This activation leads to immune cell recruitment and the formation of the membrane attack complex, causing direct cell lysis. This damage initiates an inflammatory response characterized by increased CRP production; however, it is argued that CRP and the associated inflammatory surge collectively drive the deterioration of nerve fibers alongside direct lysis. In MG, autoantibodies targeting nicotinic AChRs at the neuromuscular junction block receptor function, promote receptor internalization, and activate complement-mediated damage. This loss of functional AChRs impairs postsynaptic depolarization and muscle contraction. Additionally, it is hypothesized that a dysregulation of MMPs contributes to extracellular matrix remodeling and a localized inflammatory response. While not yet definitively proven, it is suggested that this triggers a rise in CRP levels which may, in turn, further stimulate MMP activities. This proposed reinforcing loop is a plausible model for how tissue injury and disease progression may be exacerbated beyond the initial antibody strike.

The pathophysiological mechanisms involved in GBS and MG are presented in Figure 2.

## 4. Mechanistic Insights into CRP: Pathophysiological and Clinical Implications

### 4.1. CRP in Pain Perception

Pain perception in neurological autoimmune diseases involves complex interactions forming a vicious cycle autoimmune hyperexcitability, nociceptive neuron sensitization and neuroinflammatory processes at both local and central levels [82]. Autoimmune processes trigger production of autoantibodies that directly influence nociceptive neurons by binding to ion channels and engaging Fc gamma receptors [83].

Proinflammatory mediators influence specific ion channels on nociceptors including transient receptor potential cation channel subfamily V member 1 protein (TRPV1), transient receptor potential ankyrin 1 protein (TRPA1), and voltage-gated sodium channels (Nav1.7–1.9). Upon activation, nociceptors release neurotransmitters including substance P and calcitonin gene-related peptide (CGRP) contributing to neurogenic inflammation locally. This process is however site-specific, and can spared to the vicinity of the dorsal root ganglia (DRG) and peripheral nerves, increasing additional macrophages and satellite cells activation to further increase inflammation [84].

Afari et al. [85] were the first to examine the relationship between hs-CRP and cold pain sensitivity (CPS) in female twins, demonstrating that hs-CRP levels exceeding 3.0 mg/L associated increased pain ratings at threshold and tolerance, independent of environmental factors. This association persistent for individuals not previously diagnosed with chronic pain, possibly reflecting on how subclinical inflammation can influence pain tolerance and perception.

Graham-Engeland et al. [86] examined whether pain intensity and pain interference interact with recent negative affect in relation to systemic inflammatory activity, assessed using CRP and a measurement of circulating cytokines and inflammatory profile in healthy adults. Elevated CRP levels were notably only found among subjects reporting both greater pain symptoms with recent negative effect. Physical pain with no negative effect was found to not increase circulating CRP levels. This finding suggests extending CRPs role beyond a marker for tissue damage, but also of emotional distress accompanied by those lesions. If CRP was to reliably be associated with increased emotional distress, examining patients suffering from neurodegenerative disease that also exhibit flares could prove difficult, as increased inflammatory activity markers could potentially be driven by increased stress and pain interference.

**Figure 3:**
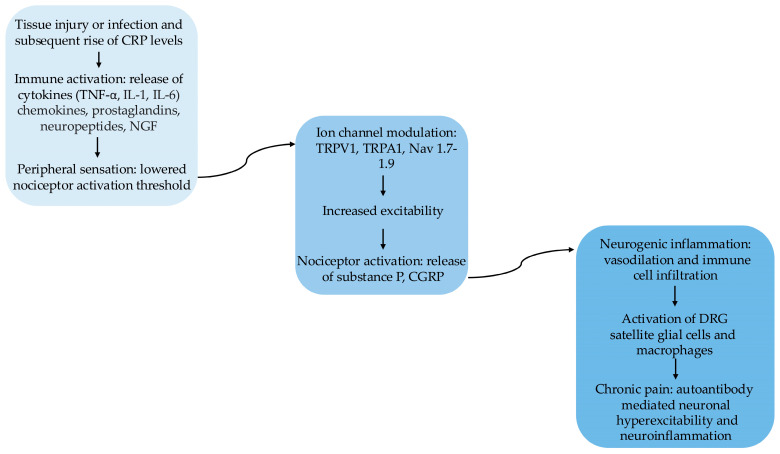
The transition from systemic immune activation to chronic pain signaling follows a multi-staged pathophysiological sequence. Acute Phase Response: following tissue injury or infection, an elevation in CRP serves as a clinical marker for the induction of pro-inflammatory cytokines (TNF-α, IL-1, IL-6) and chemokines. These mediators, along with prostaglandins and NGF, act on peripheral nociceptors to modulate ion channels such as TRPV1, TRPA1, and the voltage-gated sodium channels Nav1.7–1.9. This modulation lowers the activation threshold, resulting in heightened neuronal excitability and the release of neuropeptides (substance P, CGRP) that drive neurogenic inflammation. Proposed Chronic Mechanisms: in the theorized transition to chronic pain, sustained signaling is maintained through autoantibody-mediated neuronal hyperexcitability. This stage is characterized by glial-immune interaction within the DRG. It is proposed that the activation of satellite glial cells and resident macrophages within the DRG creates a self-perpetuating inflammatory microenvironment. This theorized “ganglionic” neuroinflammation is thought to provide the continuous amplification necessary to maintain chronic pain states.

An extensive UK biobank analysis found correlations between increased CRP levels and chronic widespread pain. Adjusting for both lifestyle factors, local and demographical factors, the association remained strong, with no sex specific associations found in headaches or facial pain. Both low and high threshold CRP values were noted in chronic pain patients, with higher values especially present in patients reporting widespread chronic pain [87]. Collectively, these findings support the broader involvement of inflammatory pathways in pain-related morbidity and highlight inflammation-related biomarkers as potential prognostic indicators and therapeutic targets (Figure 3).

### 4.2. CRP in Monitoring Disease Activity and Treatment Response

#### 4.2.1. Disorders of the CNS

Inflammation can be seen as the driver of relapses in MS, resulting from increased myelin degeneration triggered by immune cells traversing a permeable BBB. As such, regulatory agents such as vitamin D have emerged with extensive scientific backing as both therapeutic alternatives and disease monitoring markers. Vitamin D levels have been previously associated with MS-related brain atrophy and lesion burden [88,89]. Maintaining adequate levels may contribute to relapse prevention and improved clinical outcomes. However, biomarkers such as CRP and endocan (formerly endothelial cell-specific molecule-1 (ESM-1)), give more insight into the pathophysiology of relapse and remission [89].

Analysis conducted by Akil et al. demonstrated that serum levels of endocan, CRP, and neutrophil-to-lymphocyte ratio (NLR) were significantly elevated in patients with relapsing-remitting multiple sclerosis (RRMS) compared to healthy controls. Despite not adequately tracking long term disease progression or lesion burden, moderate specificity has been achieved for predicting relapses, therefore enforcing their role as markers for endothelial disfunction and inflammation [90]. However, MS progression often presents as low-grade compartmentalized inflammation associating local endothelial stress, with hsCRP demonstrating meaningful utility in measuring disease activity.

Spinal cord lesions represent a hallmark feature of NMOSD, often leading to long-term irreversible complications such as spinal cord atrophy (SCA), which substantially contributes to patient morbidity through progressive neurological disability. Retrospective analysis of 177 NMOSD patients identified SCA in 12.4%, with affected cases showing longer disease duration, higher expanded disability status scale (EDSS) scores at onset and last follow-up [91]. Prospective MRI tracking over 5 years in 56 AQP4-IgG+ patients revealed SCA progression in 28%, correlating with lesion volume and relapse frequency, while serial imaging in 102 patients showed 35% developing SCA by 3 years, with cervical atrophy rates of 1.2–2.5% annually that accelerated after multiple attacks. Elevated CRP during acute relapses strongly predicts this SCA and long-term disability accumulation, as seen with markedly higher levels in affected cohorts compared to non-SCA cases, independent of attack severity. In broader analyses, onset CRP above 10 mg/L conferred substantially elevated SCA risk, reflecting blood-spinal cord barrier disruption and axonal loss, while higher CRP consistently linked to faster EDSS progression and severe disability outcomes [92,93,94]. Thus, CRP monitoring emerges as a key prognostic biomarker alongside serial MRI for risk stratification and early intervention in relapse-prone AQP4+ patients to curb inflammation-driven atrophy.

Many disease-modifying and immunosuppressive therapies reduce systemic cytokine signaling, including IL-6 and TNF, blocking hepatocytes from effectively producing CRP and hampering its diagnostic potential in detecting subclinical inflammation [95]. As discussed prior, in MS, higher baseline CRP levels are associated with increased relapse rates and disease progression [96]. Specifically, in patients with MS or NMOSD receiving anti-CD20 therapies, such as ocrelizumab or rituximab, clinical and radiological relapses are often nearly abolished. While modest relapse potential is present, CRP levels remain deceptively low, with cytokine activity being blunted by immunosuppressive medication [96,97]. It is safer to assume in these scenarios that increased CRP values might indicate systemic inflammation or metabolic stress derived from an external source such as a urinary tract infection. In such cases Uhthoff’s phenomenon triggered by fever from a systemic infection could be mistaken with a worsening of preexisting neurological symptoms stemming from progressive neurodegeneration or a new flare [98]. CRP levels might also remain chronically elevated in relation to adiposity, as increased fat deposits also associate with increased IL-6 production [99].

#### 4.2.2. Disorders of the PNS

Altaweel et al. demonstrated how a statistically significant positive correlation was found between clinical severity, as measured by the Hughes functional grading scale scores (HFGSS), and serum CRP levels in GBS patients. Their analysis revealed that gastroenteritis, cranio-bulbar involvement, the need for mechanical ventilation, a disability score greater than 4, and absent motor and sensory responses were all significantly associated with elevated serum CRP levels exceeding 6 mg/dL [53]. This finding could suggest that CRP could be used to triage patients, estimating potential needs for further advanced critical support, immunotherapy or intense monitoring to prevent respiratory failure.

Further findings supported the previous claim, associating elevated CRP, NLR and C3 with greater initial severity in GBS patients [100], offering a possible snapshot into current disease activity and immediate complications, with limitations into medium and long term progression. The gold standard for medium term prognosis remains the modified Erasmus GBS outcome score (mEGOS) evaluating age at onset, presence of preceding diarrhea and GBS disability score at day 7 after admission [101].

In the study conducted by Yevgi, post-COVID-19 GBS patients demonstrated significantly higher HFGSS at both admission and discharge compared to non-COVID-19 GBS patients, indicating a worse functional prognosis. This specific subset of patients support the hypothesis of a dysregulated inflammatory response overreacting and producing proportionally severe damage, with the inflammatory origin of COVID-19 fundamentally changing therapeutic trajectory for these patients [102].

Although several biomarkers have been studied in GBS, research on serum inflammatory markers such as NLR, platelet-to-lymphocyte ratio (PLR), CRP, and albumin in pediatric GBS patients is even more limited. Research conducted by Ethemoglu et al. found elevated NLR and CRP levels in both adult and pediatric patients diagnosed with the disease compared to healthy controls with higher NLR and lower albumin levels correlating with worse prognosis in adults. Pediatric patients showed better functional outcomes and lower systemic inflammation, suggesting that their immune systems adapted more adequately after hospitalization, hampering future disease progression and possibly associating inflammation with worse disease progression [103].

The MG-ADL scale is a disease-specific, patient-reported outcome measure that assesses the impact of MG symptoms on daily functioning. The MG-ADL scale is widely used in clinical practice and research for monitoring disease severity, treatment response, and functional status in MG patients [104]. Both CRP and CRP-to-albumin ratio (CAR) levels show a positive correlation with disease severity measured by MG-ADL scores. As MG worsens, CRP and CAR increase, while albumin tends to decrease, reflecting a state of acute inflammation and poorer nutritional/inflammatory status [48]. In clinical practice, CRP levels may be measured along with other clinical assessments but are not routinely relied on to gauge immediate treatment response due to CRP’s non-specific nature and variability. Biomarkers more directly tied to MG pathophysiology (autoantibody titers, complement activity) and clinical scales guide treatment decisions better [105].

### 4.3. CRP as a Prognostic Tool

CAR combines CRP, an acute-phase inflammatory marker, with albumin, which reflects nutritional status and inflammatory condition. This dual aspect improves its capacity to capture the systemic response linked to disease severity and complications such as respiratory failure in GBS. CAR values above 0.21 serve as an independent predictor of respiratory failure in patients with GBS, while levels exceeding 0.19 are independently associated with poor short-term outcomes [100]. Elevated CRP levels (>5 mg/L) were significantly associated with respiratory failure and poorer prognosis in GBS, whereas fasting plasma glucose hyperglycemia (FPG) at admission alone does not predict respiratory failure. Patients with milder disease who did not require mechanical ventilation exhibited lower CRP and FPG levels, suggesting that these markers may aid in early risk stratification [106].

In a study by Çiçekli et al. [48], were observed in MG patients suggesting a potential role in disease relapse and prognosis. While albumin levels did not differ significantly between MG patients and controls at admission, lower albumin levels were associated with increased disease severity, indicating its value as a prognostic rather than diagnostic marker. CRP and CAR showed stronger correlations with both disease severity and mortality compared to albumin alone, with CAR emerging as a more reliable indicator due to its integration of inflammatory and nutritional status. Although coexisting autoimmune diseases were present in 16% of patients, consistent with existing literature, they did not significantly affect CRP, CAR, or albumin levels. In some patients with MG, various therapeutic approaches appear to be refractory, indicating resistance to standard treatments and highlighting the need for alternative or individualized management strategies [14]. Thus, further investigation into evaluative and predictive biomarkers remains essential to improve treatment responsiveness and guide personalized therapeutic strategies.

A higher CRP or CAR is associated with more severe and progressive MS subtypes as well as higher disability scores In patients with MS, elevated CRP levels correlate peripheral inflammation to central neuroaxonal damage, with CARs further stratifying MS lesions and relapse risk [96,107]. Nazeri et al. showed that a significant difference in hs-CRP levels was observed across different subgroups of active MS, associating CRP values in relapses symptom-based groups. Results suggest that CRP values could be used to differentiate between brain stem and cerebellar symptoms and pyramidal symptoms such as incontinence, associated with lower levels of CRP at onset [108].

## 5. Conclusions, Research Gaps, and Future Directions

Overall, the evidence reviewed supports a role for systemic inflammation in modulating clinical features across neuroinflammatory diseases; however, CRP should be interpreted primarily as a non-specific, downstream marker of inflammatory burden rather than a disease-driving mechanism. Across MS, NMOSD, and MG, associations between CRP and symptoms such as depression or pain are modest, context-dependent, and frequently attenuated after adjustment for metabolic comorbidities, highlighting the substantial influence of obesity, metabolic syndrome, and treatment-related factors on peripheral inflammatory markers. Notably, several studies report null or selective findings particularly for cytokines underscoring heterogeneity in inflammatory signatures and limitations in biomarker sensitivity.

Current data are further constrained by predominantly cross-sectional designs, limited longitudinal sampling, and reliance on peripheral markers that incompletely capture compartmentalized neuroinflammatory processes. While emerging interest in CRP isoforms such as mCRP offers mechanistic insight, significant methodological barriers currently limit their clinical applicability.

Future research should prioritize longitudinal approaches that integrate, confounding factors such as adiposity, chronic immunosuppressive therapy, and disease-specific biomarkers to better differentiate systemic inflammation from neuroimmune pathology.

## Data Availability

No new data were created or analyzed in this study. Data sharing is not applicable to this article.

## Abbreviations

The following abbreviations are used in this manuscript:

AChRs	Acetylcholine Receptors
AD	Alzheimer’s Disease
AKAp12	A-kinase anchoring protein 12
AMAN	Acute motor axonal neuropathy
AQP4	Aquaporin-4
BBB	Blood–brain barrier
BDNF	Brain-derived neurotrophic factor
BMI	Body mass index
C1q	Complement component 1q
CAR	CRP-to-albumin ratio
CGRP	Calcitonin gene-related peptide
CNS	Central nervous system
COX-2	Cyclooxygenase-2
CPS	Cold pain sensitivity
CRH	Corticotropin-releasing hormone
CRP	C-reactive protein
CRPtg	CRP-expressing transgenic mice
CSF	Cerebrospinal fluid
CT	Computed tomography
DRG	Dorsal root ganglion
EBV	Epstein–Barr virus
EC	Endothelial cell
EDSS	Expanded Disability Status Scale
ELISA	Enzyme-linked immunosorbent assay
EMG	Electromyography
ESM-1	Endothelial cell-specific molecule-1
ESR	Erythrocyte sedimentation rate
FcγRs	Fc gamma receptors
FPG	Fasting plasma glucose
GBS	Guillain–Barré syndrome
GGT	Gamma-glutamyl transferase
HFGSS	Hughes Functional Grading Scale Scores
HHV-6	Human herpesvirus-6
HPA	Hypothalamic–pituitary–adrenal
HRH4	Histamine H4 receptor
hs-CRP	High-sensitivity C-reactive protein
ICAM-1	Intercellular adhesion molecule 1
IgG	Immunoglobulin G
IL-10	Interleukin-10
IL-17	Interleukin-17
IL-1β	Interleukin-1 beta
IL-4	Interleukin 4
IL-6	Interleukin-6
iNOS	Inducible Nitric Oxide Synthase
MAC	Membrane attack complex
mCRP	Monomeric C-reactive protein
mEGOS	Modified Erasmus GBS Outcome Score
MG	Myasthenia gravis
MG-ADL	Myasthenia gravis activities of daily living
miRNA	Micro RNA
MMP-10	Matrix metalloproteinase-10
MMP-9	Matrix metalloproteinase-9
MMPs	Matrix metalloproteinases
MRI	Magnetic resonance imaging
MS	Multiple sclerosis
NGF	Nerve growth factor
NLR	Neutrophil-to-lymphocyte ratio
NMO	Neuromyelitis optica
NMOSD	Neuromyelitis optica spectrum disorder
NO	Nitric oxide
PC	Phosphocholine
PCD	Paraneoplastic cerebellar degeneration
pCRP	Pentameric form of C-reactive protein
PLR	Platelet-to-lymphocyte ratio
PNS	Peripheral nervous system
PPP1R15A	Protein Phosphatase 1 Regulatory Subunit 15A
QMG	Quantitative myasthenia gravis
RRMS	Relapsing-remitting multiple sclerosis
SAA	Serum amyloid A
SCA	Spinal cord atrophy
SCLC	Small cell lung carcinoma
SLE	Systemic lupus etrythematosus
SMCs	Smooth muscle cells
Th17	T-helper 17
TNF-α	Tumor necrosis factor alpha
TRPA1	Transient receptor potential ankyrin 1 protein
TRPV1	Transient receptor potential cation channel subfamily V member 1 protein
VCAM	Vascular cell adhesion molecule
